# Geographical Distribution of *Trypanosoma cruzi* Genotypes in Venezuela

**DOI:** 10.1371/journal.pntd.0001707

**Published:** 2012-06-26

**Authors:** Hernán J. Carrasco, Maikell Segovia, Martin S. Llewellyn, Antonio Morocoima, Servio Urdaneta-Morales, Cinda Martínez, Clara E. Martínez, Carlos Garcia, Marlenes Rodríguez, Raul Espinosa, Belkisyolé A. de Noya, Zoraida Díaz-Bello, Leidi Herrera, Sinead Fitzpatrick, Matthew Yeo, Michael A. Miles, M. Dora Feliciangeli

**Affiliations:** 1 Laboratorio de Biología Molecular de Protozoarios, Instituto de Medicina Tropical, Facultad de Medicina, Universidad Central de Venezuela, Caracas, Venezuela; 2 Department of Pathogen Molecular Biology, Faculty of Infectious and Tropical Diseases, London School of Hygiene and Tropical Medicine, London, United Kingdom; 3 Centro de Medicina Tropical, Facultad de Medicina, Universidad de Oriente, Puerto la Cruz, Venezuela; 4 Laboratorio de Biología de Vectores y Parásitos, Instituto de Zoología y Ecología Tropical, Facultad de Ciencias, Universidad Central de Venezuela, Caracas, Venezuela; 5 Dirección General de Salud Ambiental, Ministerio del Poder Popular para la Salud, Maracay, Venezuela; 6 Hospital Miguel Pérez Carreño, Instituto Venezolano de los Seguros Sociales, Caracas, Venezuela; 7 Sección de Inmunología, Instituto de Medicina Tropical, Facultad de Medicina, Universidad Central de Venezuela, Caracas, Venezuela; 8 BIOMED, Universidad de Carabobo, Maracay, Venezuela; Institute of Tropical Medicine, Belgium

## Abstract

Chagas disease is an endemic zoonosis native to the Americas and is caused by the kinetoplastid protozoan parasite *Trypanosoma cruzi*. The parasite is also highly genetically diverse, with six discrete typing units (DTUs) reported TcI – TcVI. These DTUs broadly correlate with several epidemiogical, ecological and pathological features of Chagas disease. In this manuscript we report the most comprehensive evaluation to date of the genetic diversity of *T. cruzi* in Venezuela. The dataset includes 778 samples collected and genotyped over the last twelve years from multiple hosts and vectors, including nine wild and domestic mammalian host species, and seven species of triatomine bug, as well as from human sources. Most isolates (732) can be assigned to the TcI clade (94.1%); 24 to the TcIV group (3.1%) and 22 to TcIII (2.8%). Importantly, among the 95 isolates genotyped from human disease cases, 79% belonged to TcI - a DTU common in the Americas, however, 21% belonged to TcIV- a little known genotype previously thought to be rare in humans. Furthermore, were able to assign multiple oral Chagas diseases cases to TcI in the area around the capital, Caracas. We discuss our findings in the context of *T. cruzi* DTU distributions elsewhere in the Americas, and evaluate the impact they have on the future of Chagas disease control in Venezuela.

## Author Summary

Chagas disease is caused by a protozoan parasite called *Trypanosoma cruzi*. *T. cruzi* infects a wide variety of mammal species in Latin America as well as man, and is spread by multiple species of blood sucking triatomine insect vectors. The presence of genetic diversity in *T. cruzi* in the Americas is well established, with six different major genetic types in circulation. The genetic diversity of *T. cruzi* in Venezuela is relatively poorly understood. In this work we present the results from the genotyping of over seven hundred isolates from 17 of the 24 states. Our dataset comprises strains isolated from wild and domestic animals, several species of triatomine vector, as well as from human Chagas disease cases, including those associated with oral transmission of *T. cruzi*. Amongst other findings, our data reveal a surprisingly high frequency of atypical genotypes in humans, particularly TcIV, which has rarely been reported. We evaluate our findings in the context of *T. cruzi* diversity elsewhere in the Americas, and assess the impact they have on the future of Chagas disease control in Venezuela.

## Introduction

*Trypanosoma cruzi*, the etiological agent of Chagas disease, infects approximately 8 million people in Latin America [1]. A further 20 million people are at risk of infection. Chagas disease is widely dispersed across 21 countries in the Americas, with a natural distribution (in wild transmission cycles) from the Southern States of the USA [2] to Central Argentina [3]. Chagas disease is a vector-borne zoonosis, and transmission is generally achieved via the infected faeces of various triatomine bug species, evacuated during a blood meal. Infection is maintained in wild transmission cycles by numerous mammalian reservoir hosts, especially opossums (*Didelphis* sp.) and armadillos (*Dasypus sp*.) [4]. Human infection occurs at foci throughout the natural distribution of *T. cruzi* where triatomines have adapted to exploit the domestic setting, but also orally (via ingestion of triatomine contaminated foodstuffs) in endemic countries, as well as via blood transfusion, organ transplantation and congenital infection in and outside of areas of traditional endemicity [1].

*T. cruzi* is likely to be ancient and indigenous to the Americas [5], [6]. Indeed, the parasite demonstrates considerable genetic diversity as initially revealed by multilocus enzyme electrophoresis (MLEE) [7]–[9]. These early studies supported the typing of the *T. cruzi* into three main groups or zymodemes, called Z1, Z2 and Z3. The implementation of further molecular techniques in combination with MLEE, allow the division of the *T. cruzi* species in six groups or discrete typing units (DTU), denoted TcI, TcIIa, TcIIb, TcIIc, TcIId and TcIIe [10]. More recently, in a meeting of experts held in Brazil [11], a new nomenclature was recommended for the intraspecific classification of *T. cruzi* discrete typing units (DTUs) into TcI, TcII, TcIII, TcIV, TcV and TcVI. However, while the *T. cruzi* DTUs are relatively genetically stable in space and time, their evolutionary, ecological and epidemiolgical significance is far from clear [4], [12]. Some limited patterns emerge (reviewed in [13]), TcII, TcV and TcVI seem largely restricted to domestic transmission cycles south of the Amazon basin, where they cause considerable human disease. TcIII is infrequent from domestic sources, strongly associated with *Dasypus novemcinctus* in terrestrial transmission cycles, and found throughout South America. TcIV is enigmatic, so far uncommon among humans, broadly limited to Amazonia and Northern South America and most commonly reported from primates; TcI is the most abundant of all *T. cruzi* lineages in silvatic transmission cycles, where it primarily infects arboreal marsupials and triatomines in lowland tropical South America and terrestrial rodents and triatomines in arid rocky ecotopes. TcI is the major cause of human disease in northern South America, but also reported from chagasic patients sporadically throughout the Southern Cone.

In Venezuela, previous studies revealed TcI in humans, triatomine bugs, wild and domestic mammals [8], [14]–[21]. *T. cruzi* genotype TcIV has been reported infecting humans, triatomine bugs and the primate *Saimiri sciureus* [8], [14]. Infection with TcIII has been found in *D. novemcinctus* and associated *Panstrongylus sp.* nymphs [22]. Overall, however, reports of *T. cruzi* DTUs in Venezuela are focal and fragmented. In the present study we report *T. cruzi* genotype data from 778 *T. cruzi* strains systematically genotyped using multiple molecular markers in our laboratory over the past 12 years. These include samples obtained from 17 of Venezuela's 24 states, acute and chronic chagasic patients, seven species of triatomine bugs, nine species of wild and domestic mammals, and representatives of silvatic, peridomestic and domestic cycles. These isolates have been classified to DTU level using biochemical and molecular techniques. Our data represent a uniquely comprehensive record of the six *T. cruzi* DTUs in Venezuela and a valuable addition to our understanding of the parasite's genetic diversity in South America.

## Materials and Methods

### Ethics statement

All procedures including use of laboratory reared mice and wild mammals, have been conducted following the regulations for the use of animals in Scientific Research included in the Code of Ethics for Life, of the National Fund for Scientific, Technological and Innovation; Ministery for Science and Technology, with the approval of the Commission on Ethics, Bioethics and Biodiversity, documents N° 7513, 07 Dec. 2007 and N° 4403, 28 Oct. 2010, based on the communication of approval of protocols by the Scientific Ethical Committee of the Institute of Tropical Medicine, Faculty of Medicine, Universidad Central de Venezuela, document N° CEC-IMT 19/2009, 13 Dec. 2005, 20 Jun. 2007, 22 Nov. 2009. In the same way, all studies involving patients and inhabitants at endemic communities, have been conducted according to the regulations for the research in humans, stated in the Code of Ethics for Life, of the National Fund for Scientific, Technological and Innovation, Ministery for Science and Technology, with the approval of the Commission on Ethics, Bioethics and Biodiversity, documents N° 7513, 07 Dec. 2007 and N° 4403, 28 Oct. 2010, based on the communication of approval by the Scientific Ethical Committee of the Institute of Tropical Medicine, Faculty of Medicine, Universidad Central de Venezuela, document N° CEC-IMT 19/2009, 20 Jun. 2007, 13 Dec. 2005, 22 Nov. 2009. All subjects were asked for their voluntary participation in this study by providing a written informed consent under the supervision and approval of the above mentioned Ethical Committees. After being sure that the informed consent was clearly understood by each individual, it was signed by every person, indicating the citizen identification card number (C.I.), in every particular case. The search for triatomine bugs inside the houses, peridomestic and surrounding areas was done with the owners/residents permission. The tests for Chagas disease on domestic mammals were carried out with the owner's permission and the procedure was approved by the Scientific Ethical Committee of the Institute of Tropical Medicine, Faculty of Medicine, Universidad Central de Venezuela, document N° CEC-IMT 19/2009, 20 Jun. 2007, 13 Dec. 2005, 22 Nov. 2009.

### Parasite isolation

*T. cruzi* isolates were obtained from chagasic outpatients from different geographical areas of Venezuela attending the Instituto de Medicina Tropical (IMT) of the Universidad Central de Venezuela (UCV) as well as chagasic patients living in rural areas of Venezuela where Chagas disease is endemic. Another group of patients were from urban areas of Caracas, the Capital city, and the neighbor State Vargas (see Table 1), all of them in the acute phase of the disease, presumably infected via oral transmission. All patient isolates were collected under informed consent following the ethical permissions of the Research Ethics Commission of the Institute of Tropical Medicine, Faculty of Medicine, Universidad Central de Venezuela. The second group of *T. cruzi* isolates was obtained from seven different species of triatomine bug and originated from insects brought to the IMT by members of the public and those obtained during fieldwork. Triatomines were identified to species level according to Lent and Wygodzinksy, 1979 [23] and in some case via molecular methods (as part of Fitzpatrick *et al*., 2008 [24]) as detailed in Table 1. A third group of *T. cruzi* isolates was found infecting nine species of wild and domestic mammal (see Table 2), captured during multiple field expeditions to endemic and urban regions.

**Table 1:** Geographical distribution of *Trypanosoma cruzi* genotypes from different hosts in Venezuela.

State	Host	TcI	TcIII	TcIV	Total	State	Host	TcI	TcIII	TcIV	Total
Anzoátegui	Human	3	0	4	7	Lara	Human	8	0	2	10
	*Panstrongylus geniculatus*	2	0	0	2		*Rhodnius prolixus* **	5	0	0	5
	*Rhodnius prolixus* *	35	0	0	35	Mérida	Human	1	0	0	1
	*Triatoma maculata*	8	0	0	8	Miranda	Human	6	0	1	7
	*Artibeus jamaicensis*	1	0	0	1		*E. mucronatus*	2	0	0	2
	*Dicotyles tajacu*	1	0	0	1		*Panstrongylus geniculatus*	170	2	0	172
	*Didelphis marsupialis*	18	0	0	18		*Panstrongylus rufotuberculatus*	1	0	0	1
	*Dasypus novemcinctus*	0	4	0	4		*Rhodnius pictipes*	1	0	0	1
	*Odocoileus virginianus*	1	0	0	1		*Triatoma nigromaculata*	1	0	0	1
Aragua	Human	0	0	1	1		*Didelphis marsupialis*	4	0	0	4
	*Panstrongylus geniculatus*	4	0	0	4		*Rattus rattus*	2	0	0	2
	*Rhodnius pictipes*	1	0	0	1	Monagas	Human	1	0	0	1
Barinas	Human	2	0	1	3	Portuguesa	Human	3	0	4	7
	*Panstrongylus geniculatus*	0	1	0	1		*Panstrongylus geniculatus*	0	1	0	1
	*Rhodnius prolixus* **	37	0	0	37		*Rhodnius prolixus* **	80	0	3	83
	*Triatoma maculata*	2	0	0	2		*Canis familiaris*	1	0	0	1
	*Didelphis marsupialis*	10	0	0	10		*Didelphis marsupialis*	8	0	0	8
	*Dasypus novemcinctus*	0	9	0	9		*Mus musculus*	1	0	0	1
	*Rattus norvegicus*	1	0	0	1	Sucre	Human	5	0	1	6
Carabobo	Human	1	0	0	1		*Panstrongylus geniculatus*	3	4	0	5
	*Panstrongylus geniculatus*	1	0	0	1		*Triatoma maculata*	2	0	0	2
	*Rhodnius prolixus* *	7	0	0	7		*Didelphis marsupialis*	4	0	0	4
	*Didelphis marsupialis*	1	0	0	1	Táchira	Human	9	0	1	10
Cojedes	Human	2	0	1	3	Trujillo	Human	5	0	1	6
	*Panstrongylus geniculatus*	2	0	0	2		*Rhodnius prolixus* **	3	0	0	3
	*Rhodnius prolixus* **	5	0	0	5		*Rattus rattus*	1	0	0	1
DC	Human	16	0	0	16		*Didelphis marsupialis*	2	0	0	2
	*Panstrongylus geniculatus*	163	0	0	163	Vargas	Human	3	0	0	3
	*Didelphis marsupialis*	1	0	0	1		*Panstrongylus geniculatus*	18	1	1	20
	*Rattus rattus*	44	0	0	44	Yaracuy	Human	4	0	0	4
	*Rhodnius prolixus* *	1	0	0	1	Total		732	22	24	778
Guárico	Human	6	0	3	9						
	*Triatoma maculata*	1	0	0	1						
	*Didelphis marsupialis*	1	0	0	1						

**: Morphological and molecular identification.

*: Morphological identification only.

**Table 2:** *Trypanosoma cruzi* genotypes from different host species in Venezuela.

Host	Species	TcI	TcIII	TcIV	Total
Bugs	*Eratyrus mucronatus*	2	0	0	2
	*Panstrongylus geniculatus*	363	9	1	373
	*Panstrongylus rufotuberculatus*	1	0	0	1
	*Rhodnius pictipes*	2	0	0	2
	*Rhodnius prolixus*	173	0	3	176
	*Triatoma maculata*	13	0	0	13
	*Triatoma nigromaculata*	1	0	0	1
Mammals	*Rattus rattus*	47	0	0	47
	*Rattus norvegicus*	1	0	0	1
	*Canis familiaris*	1	0	0	1
	*Dasypus novemcinctus*	0	13	0	13
	*Mus musculus*	1	0	0	1
	*Tayassu sp*	1	0	0	1
	*Artibeus jamaicensis*	1	0	0	1
	*Odocoileus virginianus*	1	0	0	1
	*Didelphis marsupialis*	49	0	0	49
	Human	75	0	20	95
Total		732	22	24	778

### Parasite culture and DNA isolation

Parasites were isolated via several different techniques. Briefly: parasites from chagasic patients were obtained by indirect xenodiagnosis, by hemoculture of peripheral blood, or by i.p. inoculation of Balb/c mice with peripheral blood. From wild and domestic mammals, parasites were isolated by direct xenodiagnosis, by hemoculture from peripheral or cardiac blood, or by i.p. inoculation of Balb/c mice with cardiac blood. From triatomine bugs, naturally infected or used in the xenodiagnosis, the parasites were isolated by direct culture of feces in blood agar or by i.p. inoculation of Balb/c mice with bug faeces. To achieve xenodiagnosis, we used 12 to 15 instar nymphs of *Rhodnius prolixus*, 3^rd^ or 4^th^ stage, reared in the laboratory. Initially the parasites were grown in biphasic medium blood-agar followed by culture in supplemented RPMI 1640 medium as described by Miles (1993) [25].

### Parasite phenotypic and genotyping strategies

**Figure 1:**
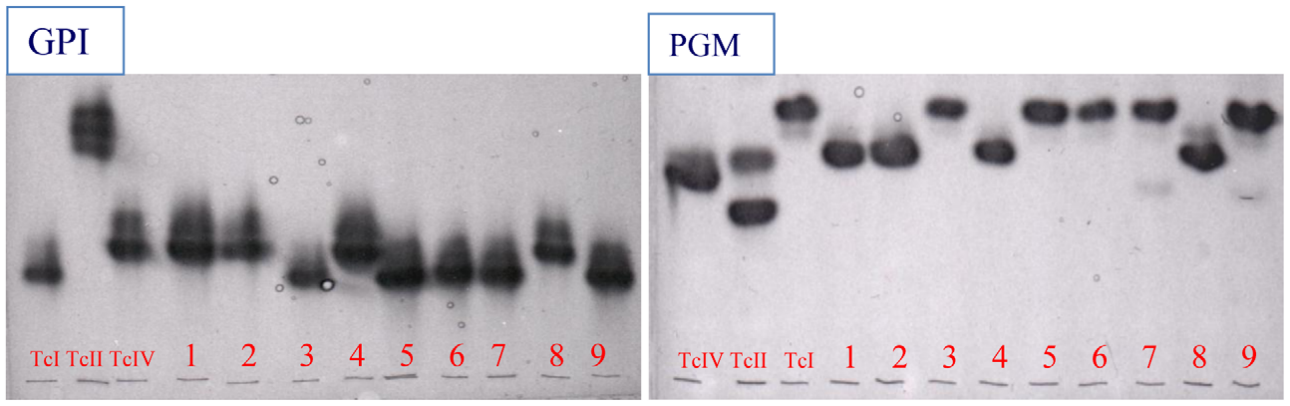
Isoenzyme profiles of GPI and PGM from representative circulating Venezuelan *Trypanosoma cruzi* strains. Loading order, left to right: TcI, WA250 cl 10B; TcII, Esmeraldo cl2; TcIV, CanIII cl 1; 1, 8839(TcIV); 2, 8196(TcIV); 3, 10141(TcI); 4, 10610(TcIV); 5, 8089(TcI); 6, 6872(TcI); 7, PGN23(TcI); 8, PGN27(TcIV); 9, PGN31(TcI).

**Figure 2:**
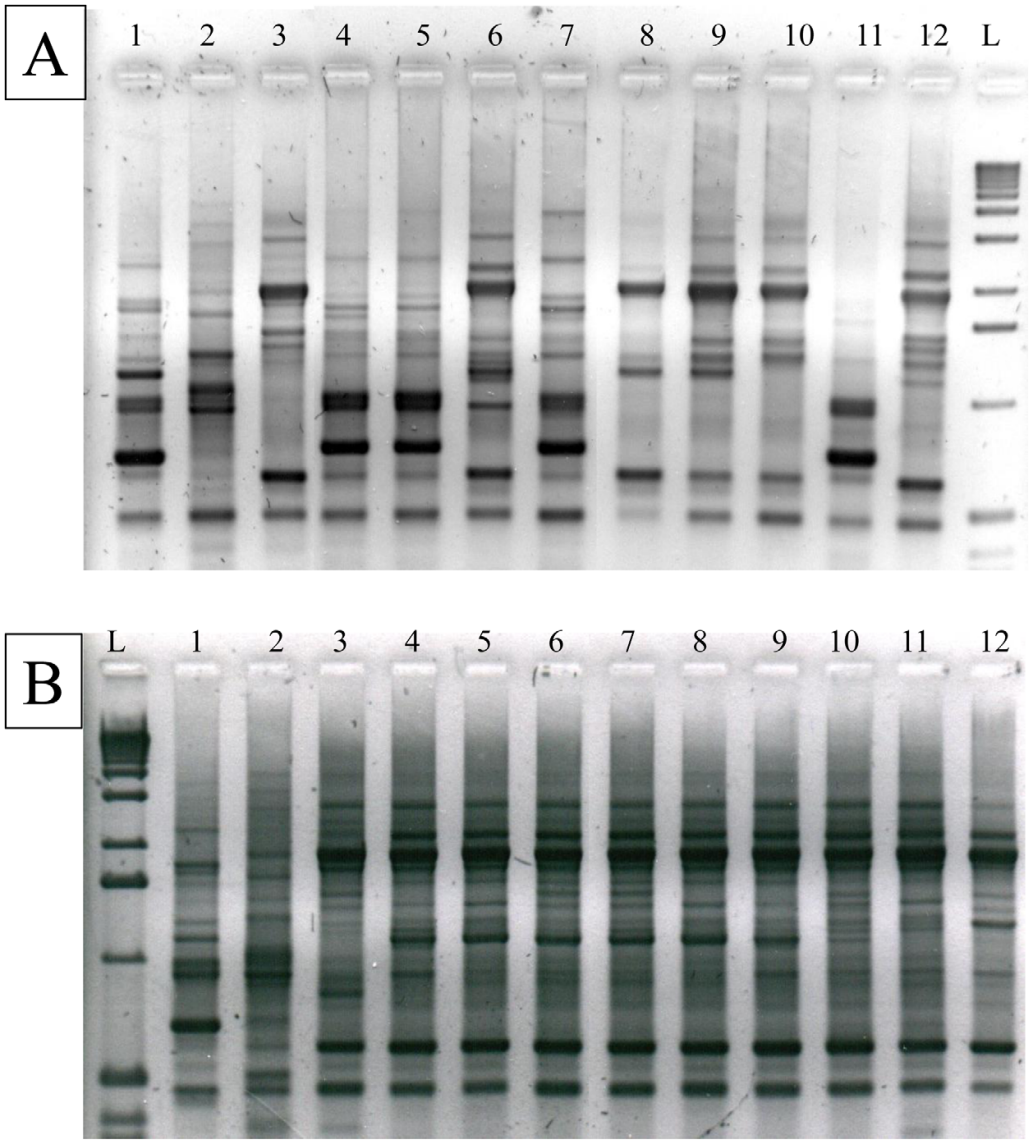
RAPD profiles of representative circulating Venezuelan *Trypanosoma cruzi* strains. Loading order, left to right **A**: 1, CanIII cl 1 (TcIV); 2, Esmeraldo cl2 (TcII); 3, WA250 cl 10B (TcI); 4, 8839(TcIV); 5, 8196(TcIV); 6, 10141(TcI); 7, 10610(TcIV); 8, 8089(TcI); 9, 6872(TcI); 10, PGN23(TcI); 11, PGN27(TcIV); 12, PGN31(TcI); L, 1 kb DNA Ladder. **B**: 1, CanIII cl1 ; 2, Esmeraldo cl2; 3, WA250 cl 10B; 4, 11932; 5, 7082; 6, 8104; 7, 7570; 8, 7780; 9, SJ1097; 10, pgn2; 11, PGCHG; 12, CD45.

**Figure 3:**
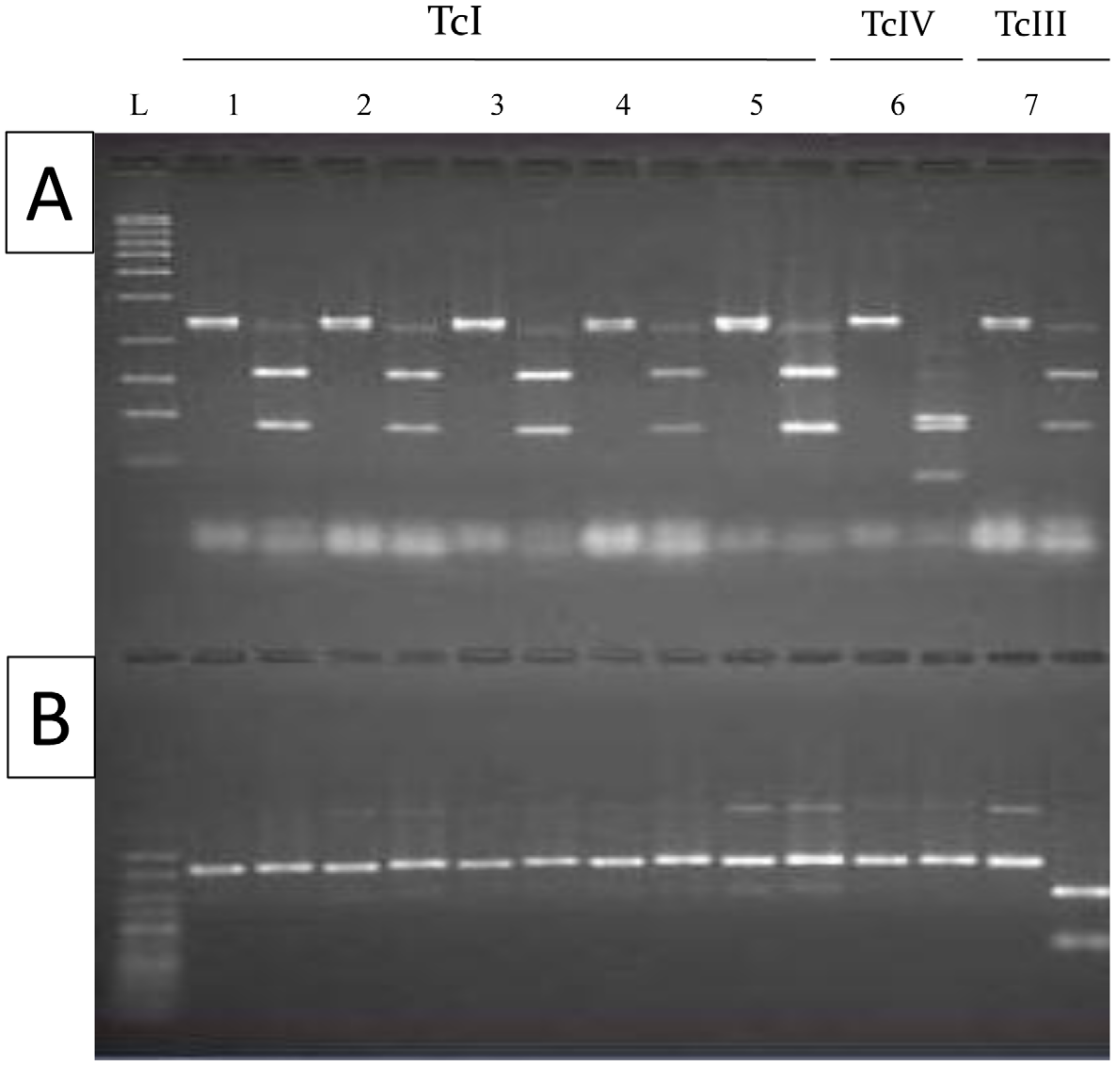
PCR-RFLP genotyping profiles of Venezuelan *Trypanosoma cruzi* strains. Each pair of lanes shows undigested PCR product followed by restriction digest products for GPI (A) and HSP60 (B). Loading order, left to right: 1, BAJV104; 2, XPMPDM5; 3, VE1003; 4, VE3303; 5, BACR104; 6, BAJT104; 7, PARAMA13; L(A), Hyperladder I (Bioline, UK); L(B), Hyperladder V (Bioline, UK).

A phenotypic analysis was initially done using the isoenzyme technique as described by Miles *et al,*. (1977) [7]. For this analysis we used phosphoglucomutase (E.C.2.7.5.1, PGM) and glucose phosphate isomerase (E.C.S.3.l.9, GPI) enzymes (Figure 1). They were examined by thin-layer starch gel electrophoresis as described by Carrasco *et. al.* (1996) [26]. Random Amplified Polymorphic DNA (RAPD) genotyping (Figure 2) was performed as in Carrasco *et al.,* (1996) [26]. PCR reactions for RAPD typing were achieved using primers A1, A2, L4 and L5 (Table 3). Each reaction took place in a 20 µL final volume containing 10 mM Tris HCl (pH 8.8) buffer, 0.2 Mm of each dNTP, 20 pg of primer, 1.0 unit of Taq DNA polymerase (Invitrogen, Brazil) and included 5 ng of whole genomic DNA. Reaction conditions were as follows: two cycles at 95°C for 5 min, 30°C for 2 min and 72°C for 1 min, 32 cycles at 95°C for 1 min, 40°C for 2 min, and 72°C for 1 min, and a final extension cycle at 72°C for 5 min. Primer sequences are listed in Table 3. PCR restriction fragment length polymorphism (PCR-RFLP) genotyping (Figure 3) targeted two loci: Glucose phosphate isomerase (*GPI*) and Heat Shock Protein 60 (*HSP60*) genes were amplified and cut using restriction enzymes *Hha*I and *Eco*RV respectively, following protocols set out in Westenberger *et al.* 2005 [27].

**Table 3:** Oligonucleotide primers employed in this study.

Method	Oligunicleotide	Secuence	Reference
RAPD	A1	5′-TCACGATGCA	Carrasco 1996
RAPD	A2	5′-GAAACGGGTG	Carrasco 1996
RAPD	L4	5′-GTGGATGCGA	Carrasco 1996
RAPD	L5	5′-AAGAGCCCGT	Carrasco 1996
PCR-RFLP	GPI(f)	5′-GGCATGTGAAGCTTTGAGGCCTTTTTCAG	Westenberger 2005
PCR-RFLP	GPI(r)	5′-TGTAAGGGCCCAGTGAGAGCGTTGGTTTGAATAGC	Westenberger 2005
PCR-RFLP	HSP60(f)	5′-GTGGTATGGGTGACATGTAC	Westenberger 2005
PCR-RFLP	HSP60(r)	5′-CGAGCAGCAGAGCGAAACAT	Westenberger 2005

All PCR products were visualised on 2.5% agarose gels (Invitrogen, USA) using appropriate molecular weight markers. Several DTU reference strains were included for comparison and are listed in Table 4.

**Table 4:** Reference strains used in this study.

*T. cruzi* I	WA250 cl 10b
*T. cruzi* II	Esmeraldo cl3
*T. cruzi* III	M6241 cl6
*T. cruzi* IV	CanIII cl1
*T. cruzi* V	Sc43 cl1;92.80 cl2
*T. cruzi* VI	CL Brener

## Results

**Figure 4:**
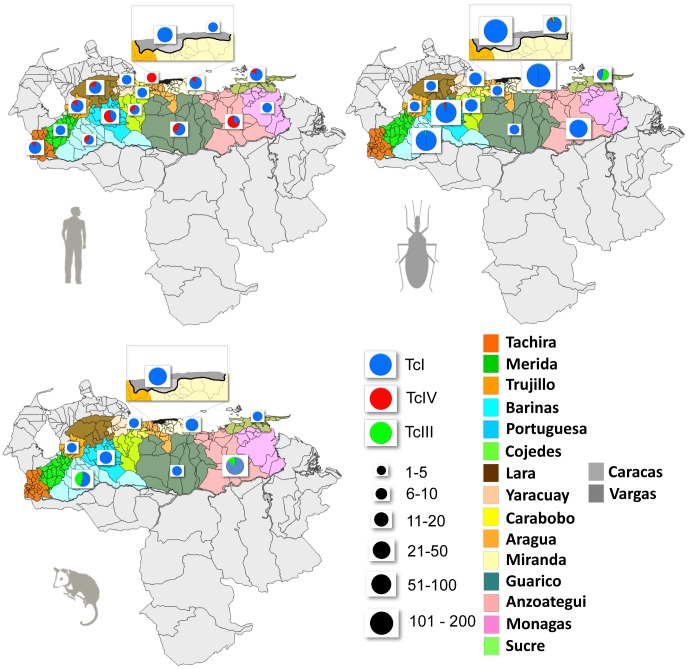
Distribution of *Trypanosoma cruzi* Discrete Typing Units (DTUs) in Venezuela. The three maps display samples collected from humans (top left), vectors (top right), and mammal reservoirs (bottom left). Pie chart area is proportional to sampling size. Pie segment colour represents DTU identity. Inset on each map shows the capital (Caracas) and surrounding states in greater detail.

In total we genotyped 778 isolates to DTU level. Images from selected electrophoretic gels for the various genotyping techniques are displayed in Figures 1–3. *T. cruzi* isolates genotyped are distributed across 17 endemic states in Venezuela. The genotype analysis of all the isolates shows that 732 belong to TcI group (94.1%); 24 isolates to TcIV group (3.1%) and 22 to TcIII group (2.8%). We recovered and genotyped 95 isolates from humans. Among these, 20 isolates were designated to TcIV (21.0%), with a further 75 typed as TcI (79.0%). Interestingly, TcIV was widely distributed across Venezuela as a secondary agent of human infection. Samples are presented by genotype, state and abundance in Figure 4 and Table 1 and 5. TcIII, although underrepresented in the total dataset (N = 22), was nonetheless conspicuous in its absence from humans. Full details of sample codes and genotypes are included in Tables S1, S2 & S3.

**Table 5:** Summary list of *Trypanosoma cruzi* genotypes by host in Venezuela.

Host	TcI	TcIII	TcIV	Total
Human	75	0	20	95
Mammals	102	13	0	115
Bugs	555	9	4	568
Total	732	22	24	778

Among nine mammal species identified with *T. cruzi* infection, TcI was the DTU most frequently encountered (102; 88.7%; Table 2 and 5). TcIII was also apparent (13; 11.3%), however, uniquely among nine banded armadillos (*Dasypus novemcinctus*). By state, TcIII was found with greatest frequency in Barinas and Anzoategui. These states were also the only ones from which *D. novemcinctus* was sampled and the presence of TcIII cannot therefore be discounted elsewhere. TcIV was absent from the infected silvatic mammals captured as part of this study, and we are thus far unable to establish the natural reservoir host of this lineage in Venezuela.

Triatomines yielded the vast majority of isolates examined (N = 568). Correspondingly, the greatest diversity of distinct *T. cruzi* DTUs (TcI, TcIII, TcIV) was also encountered among the triatomines. As previously, TcIII and TcIV represented a minority of the total number of genotypes sampled (2.3%). Among seven species of triatomine, TcIII was only recovered from *P. geniculatus*. This triatomine bug also yielded a single TcIV strain in Vargas state, close to the Distrito Capital (near Caracas), where TcI and TcIII were also identified in circulation among the same species. Interestingly, TcIV was also present among domestic *R. prolixus* in Portuguesa, where one insect showed single infection with TcIV and two others presented mixed infection of TcI and TcIV (see Table S2), in this case reflecting the high proportion of human TcIV cases in this state.

## Discussion

The 778 genotype records from humans, mammals and triatomine vectors presented in this study dramatically expand our understanding of the geographical distribution of *T. cruzi* genotypes in Venezuela.

Perhaps most significant is the frequent occurrence of TcIV among human Chagas disease cases in Venezuela. So far reports of this DTU in humans are sparse. They include at most half a dozen cases across Northern Brazil [8], [28], as well as some historical cases from Venezuela [8]. However, given the continuity of the ecotopes and major vector distributions (e.g. *R. prolixus* in Venezuela) in the areas from which these cases originate, especially the lowland Llanos region which lies between Venezuela and Colombia, we suspect that the distribution of human TcIV cases is likely underreported. We characterised TcIV from domestic *R. prolixus* at one study site in Portuguesa, and from domestic *P. geniculatus* in Vargas State (Table 1, Figure 4). As with TcI, therefore, this DTU may be actively maintained in domestic cycles in Venezuela. The risk of epizootic transmission events cannot be defined until the silvatic abundance and niche of TcIV can be established. In Brazil, and Bolivia, silvatic TcIV has been isolated primarily from primates and *Rhodnius* species triatomines [14], [28]. There are also limited records of this genotype from *Panstrongylus* species and the coati *Nasua nasua* [28]–[30]. In theory, TcIV should also be primarily located in arboreal cycles in Venezuela, associated with primates and *Rhodnius* species. Indeed, there is a single TcIV record from the squirrel monkey *Samairi scuireus* in Venezuela [14]. Targeted capture efforts should improve our understanding of enzootic TcIV in Venezuela, as well as help identify whether it shares the same risk factors for epizootic transmission as TcI (e.g. [15], [31])

*T. cruzi* is an extremely successful parasite. Evidence to support this assertion lies in its continental distribution and the sheer variety of reservoir hosts it naturally infects. TcI in Venezuela is perhaps typical of this success, with nine different species infected, including highly atypical hosts like the collared peccary (*Dicotyles tajacu*), and white tailed deer (*Odocoileus virginianus*). The epidemiological importance of atypical infections is debatable, either in terms of maintaining wild parasite transmission, or in representing a risk to human populations. Of critical relevance to human transmission in Venezuela are ecotopes dominated by palms (e.g. *Attalea* sp.), *R prolixus* vectors, and *Didelphis marsupialis* reservoir hosts. As ever, we isolated the great majority of wild TcI from *D. marsupialis*, well known as the primary host of this genotype [4] and for its tendency to aggregate around human communities [32]. Wild *R. prolixus* readily invades houses [24], establishing domestic colonies and propagating disease among rural communities. Risk factors for transmission are well established [31], and control strategies can be designed to maximise successful interruption of transmission.

TcIII, by comparison with TcI, is a less promiscuous DTU. In common with other studies through South America [22], [33], we isolated this genotype almost exclusively from *D. novemcinctus* and its associated triatomine vector *P. geniculatus*. In non-human cases, we isolated TcIII with similar global frequency to TcIV (Table 1 and 5). By comparison, no TcIII infection was observed in man, while TcIV was common. Nonetheless, we did find a TcIII infected *P. geniculatus* as a primary domestic disease vector at a peri-urban focus and it seems remarkable no TcIII was isolated from man. Similarly, TcIII is largely absent from humans throughout the rest South America, with only one confirmed report [34]. Together, these data suggest that the restricted host range of TcIII may be related to more than just transmission ecology. Detailed genetic, biochemical and biological characterisation of experimental *in vitro* and *in vivo* infections could shed light on more fundamental constraints on TcIII infectivity.

TcI transmission across Latin America is widespread [13], [35]. Several vector - reservoir host – ecological niche cliques are relevant in terms of human disease. Transmission around Caracas is an important example of the emerging importance of peri-urban transmission in the impoverished districts of several Latin American cities [17]. In Caracas rodents (*Rattus rattus*) are the primary synantropic host and *P. geniculatus* the vector (Table 1). Similarly, TcI transmission is maintained by murid rodents (although via *Triatoma* species vectors), in hyper-endemic arid sub-Andean valleys that impinge on the city of Cochabamba [36]. Peri-urban transmission in Arequipa, Peru, accounts for high levels of seropositivity even among children [37]. In this case, however, relevant reservoir host and vectors are less well characterised. Nonetheless, in Venezuela and elsewhere, control of disease transmission in an urban environment represents a very different challenge to that at rural foci. National authorities could benefit from the sharing of experience in relation to peri-urban Chagas disease control.

The great majority of human isolates from Caracas characterised in this manuscript originate from several oral Chagas disease outbreaks in the city. The largest oral outbreak so far recorded in the city occurred at a school in 2007 [21], [38]. It is thought that over 1000 were exposed, mostly children, among whom 103 developed infection and one died. Classic epidemiological approaches indentified a contaminated batch of Guava juice as the likely source, and three isolates typed from patients and nearby triatomines were TcI. Data presented here do not include the genotypes of isolates from the 2007 outbreak. Nevertheless, we have included isolates obtained from *P. geniculatus* and *R. rattus* from the site where the Guava juice was prepared, which were also TcI. In addition, we did include data from two further outbreaks, and all human genotypes also correspond to TcI. The existence of these strains, and accompanying non-human isolates from the same sites, opens the door to high resolution molecular epidemiological work to pinpoint the actual source of these oral cases. Rigorous molecular epidemiological studies can complement and enhance control recommendations for oral disease outbreaks, which are currently limited to food hygiene measures [38], to help prevent future outbreaks and perhaps shed light on the elevated case mortality rates associated [1].

Several anecdotal reports exist to suggest that human Chagas disease mega-syndromes are more common in the Southern Cone region of Latin America [12], [13]. This aspect of differential disease presentation between northern and southern South America is often circumstantially attributed to the presence of TcII, TcV and TcVI in the south [13]. Consistent with several current and historical studies, however, we observed severe cardiac forms of disease in Venezuela among TcI cases (Nessi *et al*., manuscript in preparation). To date, however, we have not detected digestive forms of the disease. Using high resolution microsatellite markers, we recently demonstrated a substantial reduction in genetic diversity among 15 TcI isolates from humans in Venezuela, by comparison to their silvatic (wild) counterparts [19]. Our analysis indicated that most human infections originate from the same genetically depauperate clade, while incursion of strains from the local silvatic environment was a far rarer event. The remaining 60 human TcI isolates that are uncharacterised by high resolution markers not only offer considerable scope to test the robustness of the TcI human clade, but, in conjunction with clinical history, may also allow us to test the strength of association between TcI sub-DTU level diversity and disease presentation. Importantly, we can confirm that a number of human TcIV cases in this study were symptomatic (Nessi *et al*., manuscript in preparation) and this DTU can be considered an epidemiologically important secondary agent of Chagas disease. High resolution analyses of TcIV isolates from human cases promise to reveal whether these isolates also represent a genetically restricted clade.

Chagas disease is potentially re-emergent in Venezuela [39]. The data presented in this manuscript are especially important to understanding the eco-epidemiology of infection locally as well as in the context on renewed efforts to interrupt transmission in rural and urban settings. Vitally, they also lay the groundwork for future, hypothesis driven research aimed at discovering the epidemiological/biological relevance of genetic diversity within the *T. cruzi* DTUs. For example, it is now technically possible to identify the sources of emergent peri-urban and oral transmission. Also, in conjunction with detailed longitudinal clinical data it may be possible to investigate the impact parasite genetic diversity has on the outcome of human disease.

## Supporting Information

Table S1 ***T. cruzi*** **genotypes from human of different States in Venezuela.**
(PDF)

Table S2 ***T. cruzi*** **genotypes from bugs of different States in Venezuela.**
(PDF)

Table S3 ***T. cruzi*** **genotypes from mammals of different States in Venezuela.**
(PDF)
