# Supplementary material for: Geographical Distribution of Trypanosoma cruzi Genotypes in Venezuela
Source: PLoS Negl Trop Dis. 2012 Jun 26;6(6):e1707. doi: 10.1371/journal.pntd.0001707 (PMC3383755; doi:10.1371/journal.pntd.0001707)
Supplement: Table S1 — T. cruzi genotypes from human of different States in Venezuela. (PDF) [file pntd.0001707.s001.pdf]

Table S1 *T. cruzi* genotypes from human of different States in Venezuela

State	Host	Strains	TcI	TcIV	Total
Anzoátegui	Human	11881 11912 **ERA JR 8215 11155**	2	4	6
Aragua	Human	**9513**	0	1	1
Barinas	Human	10775 BAJV104 **BAJT104**	2	1	3
Carabobo	Human	10868	1	0	1
Cojedes	Human	A7 11006 **8196**	2	1	3
DF	Human	11124 11841 ANT1 ANT2R ANT3 ANT4R* ANT5* ANT6 ANT7R ANT10R ANT11R ANT12R* ANT13R ANT16R* ANT17 ANT18R	16	0	16
Guárico	Human	*EPCD EPSU 7082 10801 11838 11704* **12240 10610 13067**	6	3	9
Lara	Human	11713 9639 11720 9114 11597 9878 10334 11854 **11048 8839**	8	2	10
Mérida	Human	11541	1	0	1
Miranda	Human	8089 12914 10462 11250 8104 11398 **9196**	6	1	7
Monagas	Human	11834	1	0	1
Portuguesa	Human	8396 11804 11042 **RM24 11272 XPMPB310 XPMPB910**	3	4	7
Sucre	Human	9354 8755 11274 10968 12266 **12658**	5	1	6
Táchira	Human	7570 8378 12164 11711 7780 12256 8120 12045 7388 **11717**	9	1	10
Trujillo	Human	8925 12453 9547 9010 9123 10141 **10825**	6	1	7
Vargas	Human	EL MM PJ	3	0	3
Yaracuy	Human	6872 12058 12269 11932	4	0	4
Normal latter: isolates TcI; **Bold: isolates TcIV**			75	20	95
