# Supplementary material for: Geographical Distribution of Trypanosoma cruzi Genotypes in Venezuela
Source: PLoS Negl Trop Dis. 2012 Jun 26;6(6):e1707. doi: 10.1371/journal.pntd.0001707 (PMC3383755; doi:10.1371/journal.pntd.0001707)
Supplement: Table S2 — T. cruzi genotypes from bugs of different States in Venezuela. (PDF) [file pntd.0001707.s002.pdf]

Table S2. *T. cruzi* genotypes from bugs of different States in Venezuela

State	Host	Strains	TcI	TcIII	TcIV	Total
Anzoátegui	*P. geniculatus*	V1 AMPGCHA	2	0	0	2
	R. prolixus	V4 RPAE5 RPAE2 RPNIVE1* AMMRP(NIII)E5 AMRPNIVE5* AMRP(A)E1 AMRPAE4 AMRPAE6 AMRPAE7 AMRPAE8 ACPV806 ACPV706 ACPV106 ACPV406 ACPV306 ACPV1006 ACPV606 ACPV506 ACPV1206 ACP1006 ACJV306 ACJV1506 ACJV606 ACJV406 ACJV906 ACJV1206 ACJV806 ACJV906 ACJV106 ACJV1006 ACV1106 ACJV2006 ACJV206 ACJV706	35	0	0	35
	T. maculata	V2 TMA1A TM1A TMN3E2 TMN3E5 AMTAE3 AMTMN2E1 VE2807	8	0	0	8
Aragua	*P. geniculatus*	PGN31 VE5906A VE5906B VE8608	4	0	0	4
	R. pictipes	VE2903	1	0	0	1
Barinas	*P. geniculatus*	**MUCU**	0	1	0	1
	R. prolixus	Orlando BA01104 BA03104 BA09104 BA14104 BACR104 BALN104 BA02104 BA15104 BA04SL P2V205 BrRp RPRD03 BAD1705 RBLNBA RPNM03 RPMN03A SRP2a SRP2b SRP2c LL2LA LL2LBR LL2LCR LL2LDR LL2LH RPIG03 PB3R PB5 PB4 PB7 PB1 PB5R PB6 PB8R BAOA05 1721 BALDO5	37	0	0	37
	T. maculata	BARM BACP	2	0	0	2
Carabobo	*P. geniculatus*	VP34	1	0	0	1
	R. prolixus	CALC104 CASB104 011103 010902 070214 010501 010503	7	0	0	7
Cojedes	*P.geniculatus*	VP30 VP37	2	0	0	2
	R. prolixus	RPSVCOJ TcValle TCSCII TR032 TRCIET	5	0	0	5
DF	*P. geniculatus*	VP27 VECW07 VE3405B VE1305 VE4005 VE0206 analugo VEM0107 VE1706 VE2106 VE0706 VE2406A VQC106 VQC206 VQC406 VQC506 VQC606 PG4100 VE2203 VE2603 VE6603 PGN16 PGN17 PGN23 VE0402 VE1002A VE1002B VE1003 VE1203 VE2803 VE3603 VE4203 VE5103 VE6703 VE7303 VE1502 VE2102 VE1103A VE1103B VE1504A VE1504B SJ1097 PGCHG VE1004A VE1004B VE1004C VE3204 VE2604 VE3504 VE2904 VE5204 VE5304 VE13209B VE2306 VE3006B VE3606 VE6206 VE6106 VE8306A VE8306B VE6406A VE6406B VE4206 VE6706 VQ306 VQC406 VE1007 VE1107 VE1507 VE1707 VE3307B VE3307C VE3307E VE3707 VE4207 VE5107A VE5107B VE6807 VE8307 VE8407 VE8607 VE8607A VE8407B VE8607C VE8607D VE8607E VE8707 VEClara VE0708 VE2008 VE3708 VE5208 VE5508 VE5908 VE6008 VE6408 VE7008 VE7208 VE7508A VE7508B VE7608 VE8308 VE9108 VE9408 VE9508B VE9608 VE9908 VE10008 VE10408 VE10708 VE11708 VE12008 VE12108 VE12508 VE12708 VE13408 VE13408 VE14108 VE14308 VE14308B VE15208 VE17208 VE17708 VE18708 VE32008 VE38208 VE38508 VE41108 VE51608 VE5109 VE5909B VE10309* VE14109 VE14809* VE19709 VE21109 VE22009 VE24409 VE24409D VE26009 VE27009 VE28309 VE29309 VE29509 VE29809 VE34409 VE37209 VE37209B VE37909 ANTPANS VE1009 VE48309 VE44109 VE44509 VE45609 VE49709 VE50509 VE55909 VE2905 VE58609 VE34010 VE5210 PgHC07	163	0	0	163
	R. prolixus	VE45909	1	0	0	1
Guárico	*T. maculata*	*VE5404*	1	0	0	1
Lara	*R. prolixus*	CASABONIFACIA RP18LA RPLAGUA RPLAGUA-1 RPLAGUA3	5	0	0	5
Miranda	*Eratyrus mucronatus*	EM2001 EM400	2	0	0	2

	P. geniculatus	VP31 VP33 VP28 VP29 VP35 VE48A03 VE48B03 VE2305a pgn2 PGN11 PGN12 PGN14 PGN18 PGN200 PGN700 PGN900 PGN1701 VE0702 VE0802 PGN2201 VE0902 VE2002 VE2602 VE0203 VE1303 VE2103 VE3303 VE3703 VE6303A VE6303C VE6303D VE6303E VE6403 VE6903 VE1102 VE4703 VE0204 VE0704 SLM03A SLM03B FILAS DE M VE0904 VE1404 VE1604 VE2204 VE1904 VE3404 VE1804 VE2504 VE4204 VE4804 VE4604 VE3304 VE5704 VE5804 VE2005 VE2505 VEEG05 VE2606 VE2706B VE3206 VE3306 VE3605C VEAM106VE0506 VE0806 VE1106 VE1206 VE1606 VE1806A VE0706 VE2307 VE2507A VE2507B VE2707 VE2907 VE3107 VE3207 VE3807 VE4807B VE4807C VE5007 VE5207 VE5407 VE5807 VE7507 VE8107 VE02´08 VE0508 VE1308 VE1608A VE1608B VE02608 VE2208 VE2808 VE4508 VE4708 VE4808 VE5608 VE6508 VE7908 VE7908 VE9808 VE10808A VE10808B VE11808 VE12808 VE13608A VE13608C VE13708 VE14208 VE14708 VE15008 VE15408A VE15408B VE15408D VE15408E VE15908 VE16008A VE16008B VE16108 VE16508 VE16608 VE16408A VE16408B VE16408C VE16408D VE16408E VE16808 VE17908 VE16208 VE18508 VE18608 VE18808 VE23008 VE23708 VE24508 VE31208 VE32508 VE35208 VE37008 VE38408 VE46708 VE50108 VE51908 VE24009 VE29009 VE31109b VE33409 VE37209 VE37409 VE38509 VE43209A VE50709 VE52609 VE56509 VE65309 VE55009 VE33509 VE42610 VE6010 potrerito VE021508 VE34108 VE61810 VE5510 VE2311 VE16311 VE148B11 VE4711 **VE32009** **VE3403**	170	2	0	172
	P. rufotuberculatus	VE2503	1	0	0	1
	R. pictipes	VE73310	1	0	0	1
	T. nigromaculata	VE31408	1	0	0	1
Portuguesa	*P. geniculatus*	**C21V206**	0	1	0	1
	R. prolixus	B10V1106 C4-2 TRAS 31-7-N2 PJAF-105 PMPVP25C PMPVP25BC PMPVP25BD PMPV2B PMPV3A PMPV3B PMPV3C2 PMPV1E PJRP209R PJRP109R S1.1 S1.6 S4R S10.2 S20.3 S7.1 S9.2 S13.2 S22.5AR SGG2.1 SGGALL SGP1.1 SGP1.10 SGP1.2 SGP1.20 SGP1.21 SGP1.3 SGP1.4 SGP1.5 SGP1.6 SGP1.7 SGP2.1 SFAC1.3 SFAC13 SFAC13.2 SFAC13.4 SFAC16.2 SFAC16.3 SFAC18 SFAC19.1 SFAC23.3 SFAC29 SFAC35.2 SFAC37 SFAC4.5 SFAC44 SFAC45.2 SFAC48.1 SFAC48.2 SFAC49.1 SFGARP1 SFGARP6 SFGARP8 SFGARP9 SFP10.2 SFP8.1 SFP8.2SFP8M CASAJARAMILLO TERFP TERFP1 RTERRA C42 52 53 55 56 57 58 a1329c B28V506* C32V106* B28V306* **B10RP206** B10RP106** B12RP306**	80	0	3	83
Sucre	*P. geniculatus*	EM705 EM1405 ELMACOJMC13 **EM1505** **MACOJMC14** **SPG111** **SPG3111**	3	4	0	7
	T. maculata	EM2006 EM2106	2	0	0	2
Trujillo	*R. prolixus*	S14.1 S14.2 S14.3	3	0	0	3
Vargas	*P. geniculatus*	VE1402 VE0603 VE0803A VE0803B VE0803E VE0803F VE0903 VE1703 VE1903* VE1903B VE4403 VE5503 VE2702 VE1104A* VE1104B VE1603B VE1603E VE2804 **VE1307** **PGN27**	18	1	1	20

Normal latter:isolates TcI**;** **Bold Underlined: isolates TcIII**; **Bold: isolates TcIV**

*MIX INFECTION *T. cruzi + T. rangeli*

**MIX INFECTION TcI + TcIV
