# Supplementary material for: Geographical Distribution of Trypanosoma cruzi Genotypes in Venezuela
Source: PLoS Negl Trop Dis. 2012 Jun 26;6(6):e1707. doi: 10.1371/journal.pntd.0001707 (PMC3383755; doi:10.1371/journal.pntd.0001707)
Supplement: Table S3 — T. cruzi genotypes from mammals of different States in Venezuela. (PDF) [file pntd.0001707.s003.pdf]

Table S3. *T. cruzi* genotypes from mammals of different States in Venezuela

State	Host	Strains	TcI	TcIII	Total
Anzoátegui	*D marsupialis*	AM(3-8) AM10 AM13-14 R1 R2 R2B R3 UDO UDO2 UDO3 RBHR RBCM	18	0	18
	D novemcinctus	**2MAC CACHI CACHI2 CACHIML**	0	4	4
	Dicotyles tajacu	BAQUIRO	1	0	1
	Odocoileus virginianus	VENADO	1	0	1
	Artibeus jamaicensis	VPMT	1	0	1
Barinas	*D novemcinctus*	**M5 M6 M8 M10 PARAMA3 PARAMA6 XPARAMA25 XPARAMA26 PARAMA34**	0	9	9
	D marsupialis	M7 M12 M13 M14 M15 M16 M18 P39 P40 P41	10	0	10
	Rattus norvegicus	DOM1	1	0	1
Carabobo	*D marsupialis*	DM30	1	0	1
DF	*D marsupialis*	CO57	1	0	1
	Rattus rattus	R4 R6 R8 R11 R22 R24 R26 R27 R34 R37 R42 R45 R46 R47 R50 R53 R54 R57 R58 R67 R68 R69 R71 R72 R75 R78 R79 R81 R83 R84 R88 R92 R93 R96 R97 R99R R100 R101 R102 R103 R104 R106 R108 R114	44	0	44
Guárico	*D marsupialis*	XDMG106	1	0	1
Miranda	*D marsupialis*	IN16 CD45 CO75 CO79	4	0	4
	Rattus rattus	CO22 CO84	2	0	2
Portuguesa	*Cannis familiaris*	Perra Mariposa	1	0	1
	Mus musculus	PMPMM2	1	0	1
	D marsupialis	DMJ0108 PJDM109 PJDM209 PJDM309 XPJDM5 XPMPDM2 XPMPDM3 XPMPDM5	8	0	8
Sucre	*D marsupialis*	DMSU8 DMSU2 SMSUC SM606	4	0	4
Trujillo	*Rattus rattus*	RR5	1	0	1
	D marsupialis	DM1* DM4	2	0	2

Normal latter: isolates TcI**;** **Bold Underlined: isolates Tc3**
*MIX INFECTION *T. cruzi* + *T. rangeli*
